# Oxytocin maintains lung histological and functional integrity to confer protection in heat stroke

**DOI:** 10.1038/s41598-019-54739-1

**Published:** 2019-12-05

**Authors:** Cheng-Hsien Lin, Cheng-Chia Tsai, Tzu-Hao Chen, Ching-Ping Chang, Hsi-Hsing Yang

**Affiliations:** 1 Department of Medicine, Mackay Medical College, New Taipei City, Taiwan; 2 Department of Surgery, Mackay Memory Hospital, Taipei, Taiwan; 3 Department of Medical Research, Chi Mei Medical Center, Tainan, Taiwan; 4 Department of Intensive Care Medicine, Chi Mei Medical Center, Tainan, Taiwan; 5 Department of Biotechnology and Food Technology, Southern Taiwan University of Science and Technology, Tainan, Taiwan

**Keywords:** Environmental impact, Molecular medicine

## Abstract

Oxytocin (OT) has been reported to have a protective effect in lipopolysaccharide-induced experimental acute lung injury (ALI). However, its role in heat stroke-related ALI has never been investigated. Herein, we aimed to explore the therapeutic effects and potential mechanism of action of OT on heat-induced ALI. Rats were treated with OT 60 min before the start of heat stress (42 °C for 80 min). Twenty minutes after the termination of heat stress, the effects of OT on lung histopathological changes, edema, acute pleurisy and the bronchoalveolar fluid levels of inflammatory cytokines and indicators of ischemia, cellular damage, and oxidative damage were assessed. We also evaluated the influence of OT pretreatment on heat-induced hypotension, hyperthermia, ALI score, and death in a rat model of heat stroke. The results showed that OT significantly reduced heat-induced lung edema, neutrophil infiltration, hemorrhage score, myeloperoxidase activity, ischemia, and the levels of inflammatory and oxidative damage markers in bronchoalveolar lavage fluid. The survival assessment confirmed the pathophysiological and biochemical results. An OT receptor antagonist (L-368,899) was administered 10 min before the OT injection to further demonstrate the role of OT in heat-induced ALI. The results showed that OT could not protect against the aforementioned heat stroke responses in rats treated with L-368,899. Interestingly, OT treatment 80 min after the start of heat shock did not affect survival. In conclusion, our data indicate that OT pretreatment can reduce the ischemic, inflammatory and oxidative responses related to heat-induced ALI in rats.

## Introduction

Oxytocin (OT) is a neuropeptide of 9 amino acids synthesized in the paraventricular and supraoptic nuclei of the hypothalamus^1,2^. OT has activity in uterine contraction during parturition and the milk-ejection reflex during lactation and has beneficial effects in reducing anxiety and stress disorders^2^ and hepatic^3^, cardiac^4^, myocardial^5^, renal^6^, and cerebral ischemia/reperfusion injury^7,8^. Since OT possesses anti-inflammatory, antiapoptotic and antioxidant properties^9,10^, it is hypothesized to be a beneficial agent for reducing ischemia/reperfusion injury.

Studies have indicated that ischemia/reperfusion injury in several vital organs, including the intestines, lungs, and brain, is the leading cause of heat stroke^11–14^. Patients with heat stroke present with acute lung injury (ALI) characterized by lung edema, neutrophil infiltration and hemorrhage^14^. Recent reports have also noted ALI in the lungs of baboons^15^ and rats^16^ during heat stroke. A more recent report demonstrated that OT protects against ALI in mice treated with lipopolysaccharide (LPS)^17^. Since heat stroke resembles LPS-induced sepsis in many aspects^15,16^, OT pretreatment may protect against heat-induced ALI.

To address this hypothesis, we assessed the temporal profiles of edema (e.g., lung water content and Evans blue dye extravasation), acute pleurisy (e.g., exudate volume and polymorphonuclear [PMN] cell accumulation), and the levels of ischemia indicators (e.g., glutamate and lactate/pyruvate ratio), cellular damage indicators (e.g., glycerol and lactate dehydrogenase), inflammatory cytokines (e.g., tumor necrosis factor-alpha [TNF-α], interleukin-1β [IL-1β], IL-6, IL-10, and myeloperoxidase [MPO] activity), and oxidative damage indicators (e.g., nitric oxide [NO] and dihydroxybenzoic acid [DHBA]) in the bronchoalveolar fluid (BALF) of rats pretreated or post-treated with OT. We also evaluated the influence of OT therapy on heat-induced hypotension, hyperthermia, ALI score, and death in a rat model. The OT receptor antagonist (L-368,899) was given 10 min before OT to further demonstrate the role of OT in heat-induced ALI in rats^17^.

## Results

### OT reduced heat-induced death, hyperthermia and hypotension

**Figure 1:**
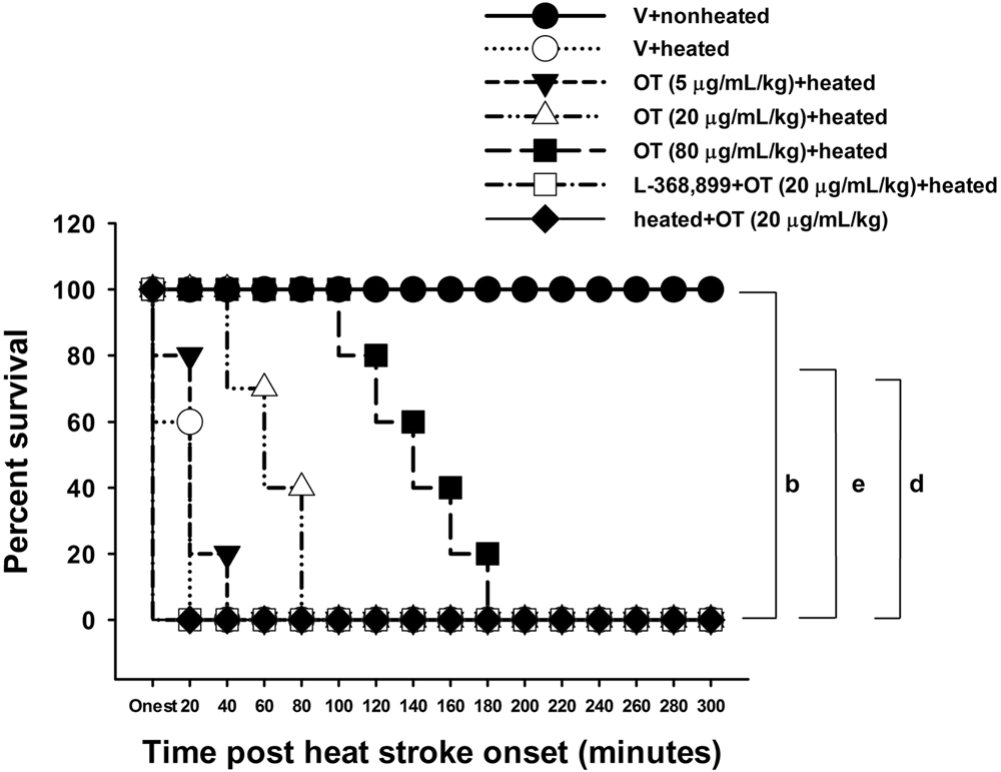
Percent survival determined by Kaplan-Meier analysis in each group (n = 10 per group). ^b^P < 0.01, V + heated group vs. V + nonheated group; ^e^P < 0.01, OT (20 μg/mL/kg) or OT (80 μg/mL/kg) + heated group vs. V + heated group; ^d^P < 0.05, L-368,899 + OT + (20 µg/mL/kg) + heated group vs. V + OT (20 µg/mL/kg) + heated group. All the rats in the V + nonheated group survived for more than 300 min. Please see the explanations of the groups in the Methods section. OT = oxytocin.

The percent survival was first analyzed to investigate the ability of OT pretreatment to reduce the lethality of experimental heat stroke. Compared to the heated controls, the heated rats pretreated with OT (5 μg, 20 μg, or 80 μg/mL/kg body weight, i.v.) showed a significant dose-dependently increase in percent survival (Fig. 1). However, when OT (20 µg/mL/kg body weight, i.v.) was administered to heated rats at the end of the heat stress period (80 min after the start of heat stress), the increase in percent survival was not significant (Fig. 1). Additionally, the beneficial effects of OT pretreatment were significantly reduced by L-368,899 treatment 10 min before OT injection, as demonstrated in L-368,899 + OT + heated rats (Fig. 1).

**Figure 2:**
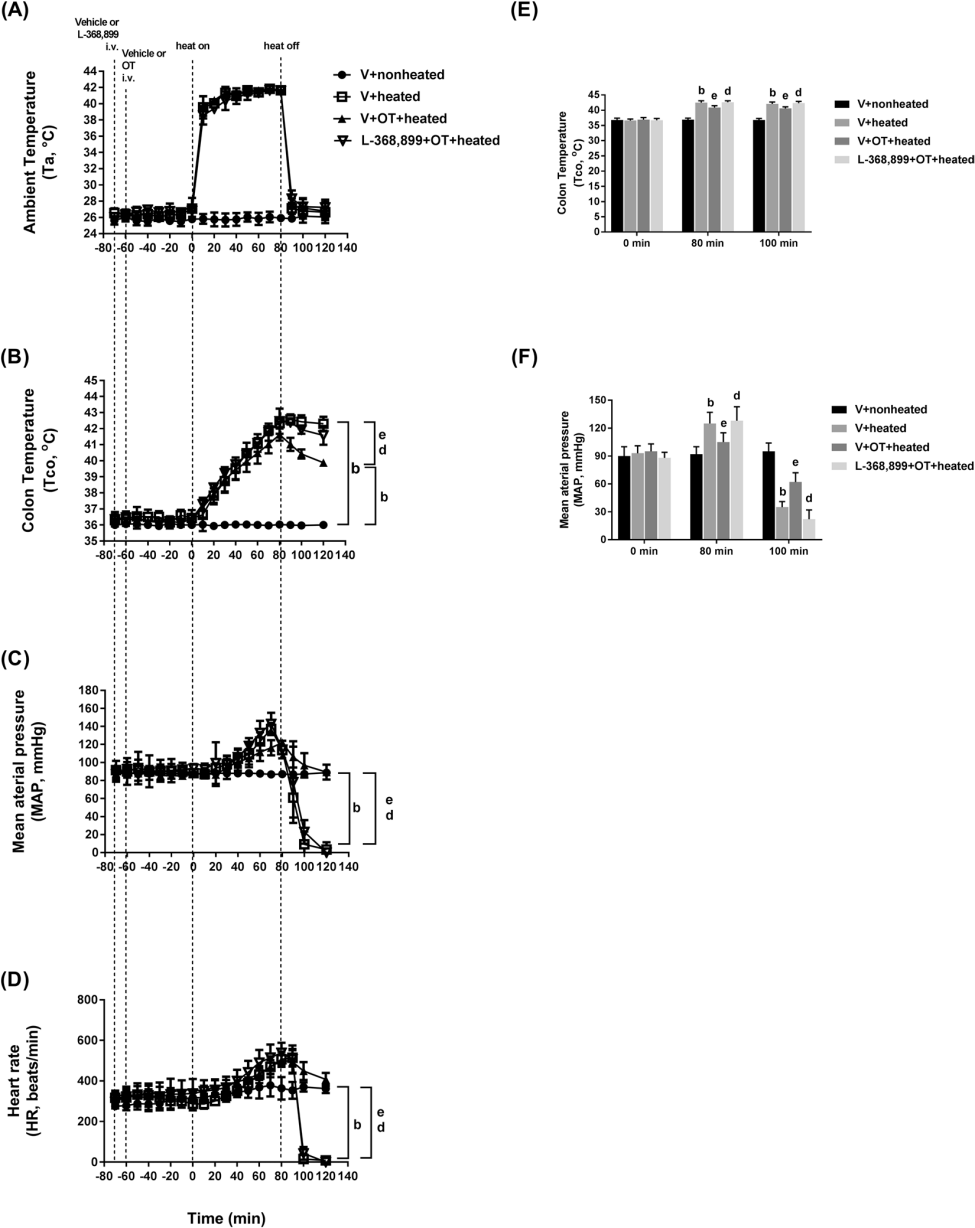
Changes in ambient temperature (**A**, Ta), colon temperature (**B**, Tco), MAP (**C**) and heart rate (**D**, HR) in the V + nonheated, V + heated, V + OT + heated, and L-368,899 + OT + heated groups. The Tco (**E**) and MAP (**F**) were obtained 0, 80, and 100 min after the initiation of heat exposure (ambient temperature in nonheated controls) in heatstroke rats. All heated groups were exposed to heat (42 °C) for exactly 80 min and were then allowed to recover at room temperature (26 °C). Data are presented as the mean ± SD of 10 rats per group. ^b^P < 0.05 compared with the V + nonheated group; ^e^P < 0.05 compared with the V + heated group; ^d^P < 0.05 compared with the V+ OT + heated group. Please see the explanations of the groups in the Methods section. V = vehicle; i.v. = intravenous.

One hundred minutes after the start of heat stress (or 20 min after termination of the 80-min heat shock), the vehicle-treated heated rats had a significantly higher core temperature (Tco) (42.2 °C vs. 36 °C) and a significantly lower mean arterial pressure (MAP) (28 mmHg vs. 86 mmHg) and HR (~150 beats/min vs. ~500 beats/min) than the vehicle-treated nonheated rats (Fig. 2). However, compared to the vehicle (V) + heated group, the OT (20 μg/mL/kg) + heated group had a significantly lower Tco (40.5 °C vs. 42.2 °C) but a significantly higher MAP (90 mmHg vs. 28 mmHg) and HR (410 beats/min vs. 135 beats/min). Again, the beneficial effect of OT treatment prior to heat stroke was significantly reduced by L-368,899 pretreatment (Fig. 2). It can be seen from Fig. 2 that at 80 min after heat shock, compared to the V + heated group, the V + OT + heated group had a slight change in both MAP and heart rate. However, at 100 min post-heat shock, the OT + heated group had significantly higher values of both MAP and heart rate than did the V + heated group.

### OT alleviated heat-induced ALI

**Figure 3:**
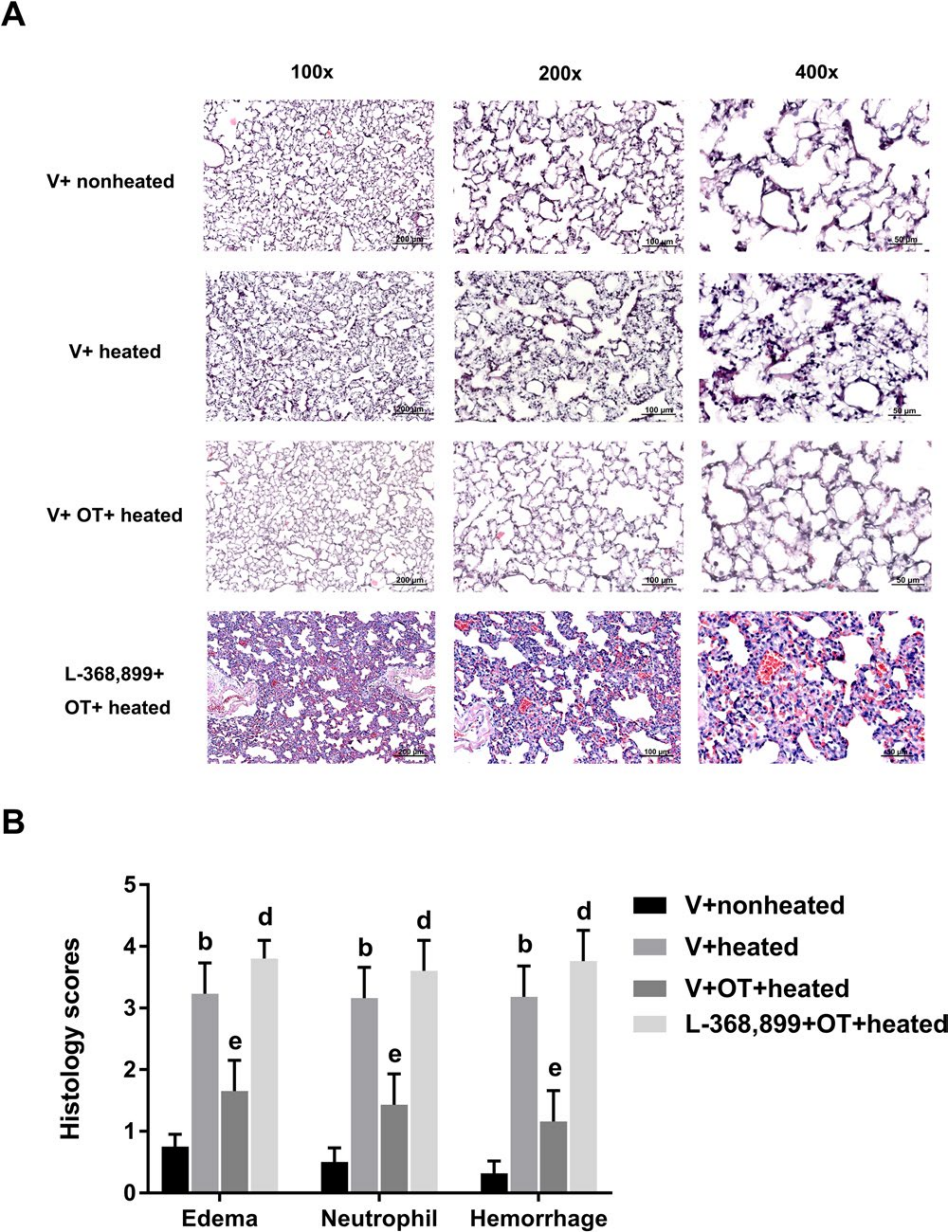
Lung edema, neutrophil infiltration and hemorrhagic scores in different groups of rats. (**A**) Representative microscopic images of the lungs from rats in the V + nonheated, V + heated, V + OT + heated and L-368,899 + OT + heated groups. (**B**) Data are presented as the mean ± SD (n = 10). ^b^P < 0.05, V + heated group vs. V + nonheated group; ^e^P < 0.05, V + OT + heated group vs. V + heated group; ^d^P < 0.05 compared to the L-368,899 + OT + heated group.

The pulmonary edema, neutrophil and hemorrhage scores were significantly higher in the V + heated group than in the nonheated control group (Fig. 3). The heat-induced increases in lung edema, neutrophil and hemorrhage scores were all significantly reduced by OT pretreatment, as demonstrated by the V + OT + heated group (Fig. 3). However, the beneficial effects of OT in reducing ALI were significantly reversed by pretreatment with the OT antagonist L-368,899 (Fig. 3).

### OT reduced the heat-induced increases in BALF levels of PMN cell number and proinflammatory cytokines and in lung MPO activity

Compared with the nonheated control group, the V + heated group had significantly higher exudate volume, PMN cell number and proinflammatory cytokine levels in BALF and lung MPO activity (Table 1). Compared with the V + heated group, the V + OT + heated group had significantly lower exudate volume, PMN cell number and proinflammatory cytokine levels in BALF and lung MPO activity (Table 1). The beneficial effects of OT in reducing acute inflammatory responses to heat stress were all attenuated by L-368,899 pretreatment (Table 1).

**Table 1:** Exudate volume, PMN cell number, MPO activity in lung tissue, and TNF-α, IL-1β, IL-6 and IL-10 levels in BALF from the V + nonheated, V + heated (42 °C for 80 min), V + OT (20 µg/kg) + heated and L-368,899 + OT (20 µg/kg) + heated groups.

Parameters	V + nonheated	V + heated	V + OT + heated	L-368,899 + OT + heated
1. Exudate volume (mL)	0.3 ± 0.1	3.2 ± 0.2^b^	1.5 ± 0.2^e^	3.4 ± 0.3^d^
2. PMN cells (million cells/rat)	15 ± 6	174 ± 8^b^	82 ± 6^e^	181 ± 9^d^
3. MPO activity (μg/mg protein)	97 ± 14	326 ± 17^b^	133 ± 19^e^	329 ± 18^d^
4. TNF-α (pg/mL)	9 ± 2	69 ± 4^b^	22 ± 5^e^	72 ± 5^d^
5. IL-1β (pg/mL)	18 ± 3	77 ± 5^b^	24 ± 4^e^	80 ± 6^d^
6. IL-6 (pg/mL)	12 ± 1	78 ± 3^b^	26 ± 3^e^	79 ± 4^d^
7. IL-10 (pg/mL)	31 ± 5	57 ± 6^b^	123 ± 8^e^	53 ± 5^d^

Data are presented as the mean and SD for ten rats/group. ^b^P < 0.05 compared with the V + nonheated group; ^e^P < 0.05 compared with the V + heated group; ^d^P < 0.05 compared with the V + OT + heated group.

Rats were sacrificed 100 min after the initiation of heat stress by an overdose of anesthetic, and the samples were collected for biochemical determination.

### OT downregulated the heat-induced increase in BALF levels of cellular ischemia and damage markers and nitrogen and oxygen free radical species

Compared with the V + nonheated group, the V + heated group had significantly higher BALF levels of cellular ischemia indicators (e.g., glutamate and lactate/pyruvate), cellular damage indicators (e.g., glycerol) and nitrogen and oxygen radical species (e.g., NO metabolites and DHBA) (Table 2). The heat-induced lung ischemia and oxidative damage were significantly alleviated by OT pretreatment. Again, L-368,899 significantly reversed the beneficial effects of OT (Table 2).

**Table 2:** Lactate/pyruvate ratio and the levels of glutamate, glycerol, lactate dehydrogenase, NO metabolites, and DHBA in BALF from the V + nonheated, V + heated (42 °C for 80 min), OT (20 μg/kg) + heated and L-368,899 + OT (20 μg/kg) + heated groups.

Parameters	V + nonheated	V + heated	V + OT + heated	L-368,899 + OT + heated
1. Glutamate (% of baseline)	100 ± 18	422 ± 26^b^	153 ± 23^e^	430 ± 27^d^
2. Lactate/pyruvate ratio (% of baseline)	100 ± 9	199 ± 12^b^	124 ± 11^e^	207 ± 14^d^
3. Glycerol (% of baseline)	100 ± 8	185 ± 9^b^	112 ± 6^e^	199 ± 10^d^
4. Nitric oxide metabolites (% of baseline)	100 ± 11	453 ± 24^b^	149 ± 19^e^	463 ± 25^d^
5. 2,3-Dihydroxybenzoic acid (% of baseline)	100 ± 14	207 ± 22^b^	138 ± 16^e^	215 ± 23^d^

The results are presented as the mean ± SD (N = 10). ^b^P < 0.05 compared with the V + nonheated group; ^e^P < 0.05 compared with the V + heated group; ^d^P < 0.05 compared with the V + OT + heated group.

Rats were sacrificed 100 min after the initiation of heat stress by an overdose of anesthetic, and the samples were collected for biochemical determination.

### OT decreased heat-induced lung edema

**Figure 4:**
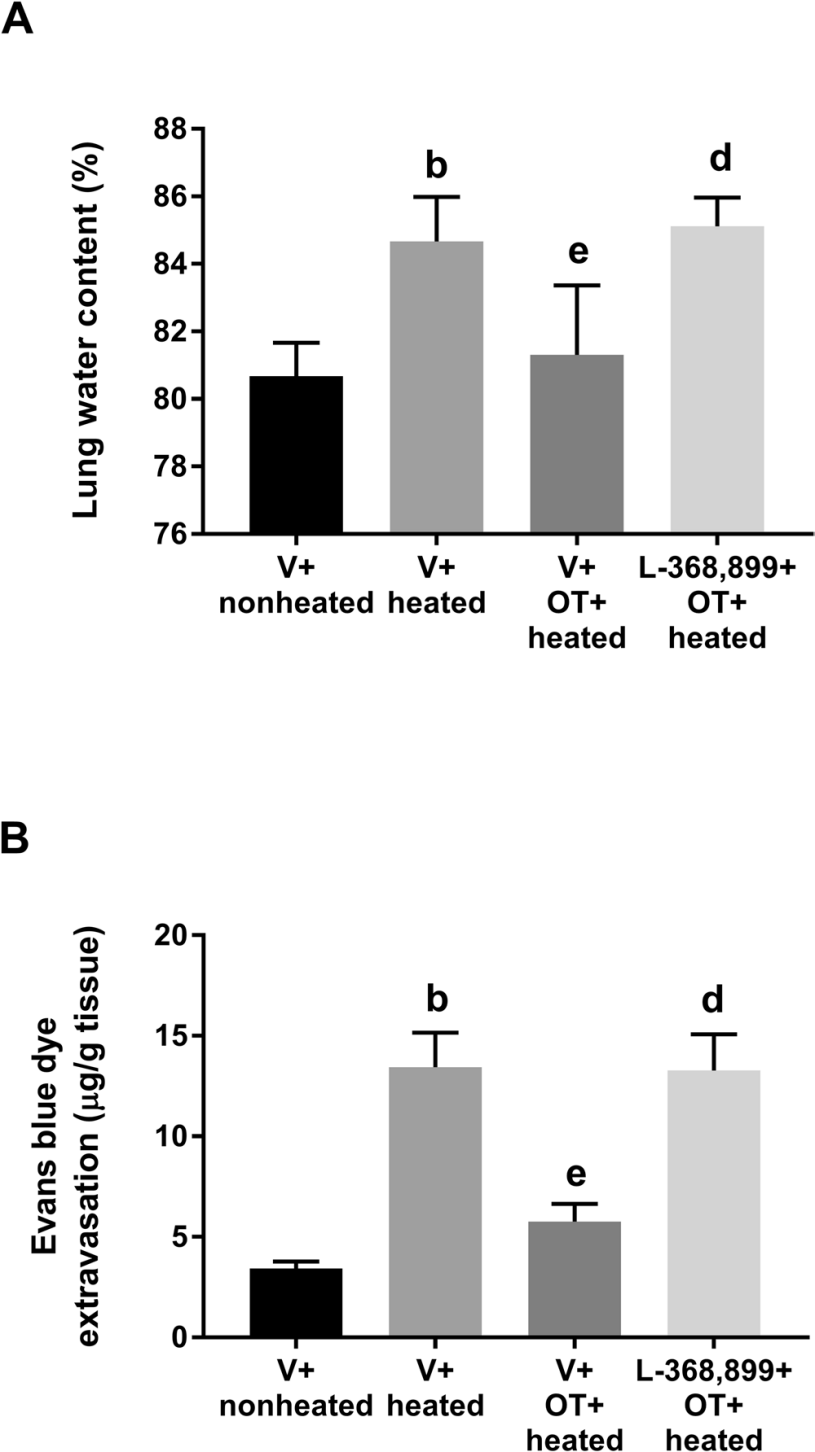
Lung water content (**A**) and Evans blue dye extravasation (**B**) in different groups of rats. All heated groups were exposed to heat for exactly 80 min and then allowed to recover at room temperature. Data are presented as the mean ± SD of 10 rats per group. ^b^P < 0.05, V + heated group vs. V + nonheated group; ^e^P < 0.05, V + OT + heated group vs. V + heated group; ^d^P < 0.05, L-368,899 + OT + heated group vs. V + OT + heated group.

The lung water content due to edema was higher in the V + heated group than in the V + nonheated group (P < 0.05, Fig. 4A). The V + OT + heated group had a significantly lower lung water content than the V + heated group (P < 0.05, Fig. 4A). Evans blue dye extravasation assays were conducted to evaluate blood-lung barrier permeability. The V + heated group had more extravasated dye in the lung specimen than did the V + nonheated group (Fig. 4B). Treatment with OT preserved blood-lung barrier integrity in the V + OT + heated group, as shown by the significant reduction in Evans blue dye extravasation compared to the V + heated group (P < 0.05, Fig. 4B). Again, the beneficial effects of OT were significantly reduced by L-368,899 treatment (P < 0.05, Fig. 4).

## Discussion

Accumulated evidence indicates that severe heat stress causes splanchnic vasoconstriction and arterial hypotension and results in bacterial translocation from the intestine to the blood stream^18^. Increased plasma endotoxin levels promote the pulmonary recruitment and activation of PMN cells^16,19^ and the overproduction of pulmonary proinflammatory cytokines^20,21^ and reactive nitrogen and oxygen species^22,23^. Indeed, our present results confirmed that rats with heat stroke displayed (i) hyperthermia and hypotension; (ii) acute pleurisy and lung edema; (iii) increased lung edema, neutrophil infiltration and hemorrhage scores; (iv) pulmonary inflammatory, ischemic and oxidative injury; and (v) decreased survival. The present study revealed a novel finding that pretreatment with OT increased survival, decreased both hyperthermia and hypotension, decreased acute pleurisy, and decreased acute lung edema, ischemia, and inflammatory and oxidative damage in heated rats. An OT receptor antagonist (L-368,899) was administered 10 min before the OT injection to further demonstrate the role of OT in heat-induced ALI; under these conditions, OT could not alleviate the aforementioned ALI. Interestingly, treatment with OT at the end of the heat shock period did not increase survival. In conclusion, our data suggest that OT pretreatment can reduce heat-induced ALI responses in rats.

Heat stroke reactions resemble LPS- or endotoxin-induced septic shock in many aspects^24^. Indeed, OT protects against ALI caused by endotoxin-induced septic shock in rats^4,25,26^. OT alleviates hepatic^3^, cardiac^4,5^, renal^6^, gastric^27^ and cerebral^1^ ischemia/reperfusion injury as well as septic shock^3,6,27^ in rodents by acting as an anti-inflammatory and/or antioxidant agent. OT reduced the inflammatory responses related to LPS-induced ALI in a mouse model^17^. Specifically, OT decreased the release of IL-1β and IL-6 but increased the release of anti-inflammatory cytokines such as IL-4 and IL-10. In ALI, neutrophils are the earliest immune cells recruited to the lungs. MPO activity is an indicator of neutrophil accumulation in tissue. LPS-induced lung histopathological injury, edema, neutrophil infiltration and MPO activity were all significantly decreased by OT. Moreover, the anti-inflammatory effects of OT in LPS-induced ALI were effectively blocked by the OT receptor antagonist L-368,899.

OT also exerts neuroprotective^28^, cardioprotective^4^ and pulmonary protective^17^ effects during ischemia/reperfusion injury, but treatment must be initiated prior to the induction of injury. The results presented herein show that OT administration before the induction of regional ischemia and reperfusion reduced the extent of tissue injury by a mechanism involving the activation of OT-specific receptors. Additionally, in our study, lung injury was not reduced in heated rats treated with OT after the onset of heat stroke, indicating that the induction of new genes is not necessary for the pulmonary protective effect of OT.

Although OT and OT receptors exert neuroprotective, cardioprotective and pulmonary protective activities, the variety and multiplicity of effects induced by OT and its receptors suggest that their roles in physiology and pathology are not fully understood^29^; the best-known and most well-established roles are the stimulation of uterine contraction during parturition and milk release during lactation. Activation of the OT receptor causes myometrial contractions. Atosiban is currently the only OT receptor antagonist that is available as a tocolytic. L-368,899, a potent non-peptide OT antagonist, inhibited spontaneous nocturnal uterine contractions in pregnant rhesus monkeys and blocked OT-stimulated postpartum uterine activity in women^30^; it also altered maternal and sexual behavior in one adult monkey^31^. Although several studies tend to indicate that OT combined with OT receptors have promise to improve the management of ALI^17^, myocardial injury^4^ and cerebral ischemia^8^, it may exert its actions via the neurohumoral system and different variety of receptors other than OT receptors. For example, oxytocin may pass the blood-brain barrier to exert its action^32,33^. Oxytocin in high concentrations may induce pressor effects by binding to vasopressin (V1a) receptors^34^. The vasoconstriction effect of OT of more than 10^−6^ M could be completely blocked by V1 receptor antagonists^35^. In contrast, OT inhibits the rat medullary dorsal horn nociceptive transmission through OT but not V1a receptors^36^. Thus, the exact mechanisms behind the pulmoprotective effects of OT in heated rat remain to be explored in future investigation.

We noted that OT protects against heat-induced hypotension, which suggests the potential for OT as a preventive strategy for heat stroke. Both patients^37^ and rodents^38^ display arterial hypotension during heat stroke. A more recent report showed that arterial hypotension occurs at the onset of severe heat stroke due to decreased cardiac mechanical efficiency and arterial elastance^39^. Thus, OT preconditioning might attenuate arterial hypotension in rats experiencing heat stroke by the same type of mechanism.

Compared with the normothermic control rats in the present study, the vehicle-treated heated rats had more severe cellular ischemia (e.g., glutamate and lactate/pyruvate ratio) and damage (e.g., glycerol); higher levels of prooxidant enzymes (e.g., lipid peroxidation and glutathione oxidation markers), proinflammatory cytokines (e.g., IL-1β, IL-6 and TNF-α) and NO in BALF; and greater PMN leukocyte accumulation (e.g., MPO activity) in lung tissue. However, indicators of pulmonary antioxidant defense (e.g., glutathione peroxidase and glutathione reductase) were lower. Thus, it appears that OT preconditioning confers protection against heat stroke in rats by attenuating heat-induced inflammatory, ischemic and oxidative damage to the lungs.

The present study demonstrated that OT binding to OT receptors has a protective effect against heat-induced ALI by inhibiting the ischemic, oxidative and inflammatory responses in a rat model. The mechanism might involve TLR4/NLRP3/NF-κB, Bax/p53, Bcl-2^40,41^, type A gamma-aminobutyric acid receptor^28^, calpain-1^7^, and carbonic anhydrase and acetylcholinesterase enzymes^42^.

Environmental heat stress increases cutaneous blood flow and metabolism and progressively decreases splanchnic blood flow^24^. Tissue ischemia, rather than hyperthermia, is the main cause of heat stroke. In the present study, OT attenuated ALI in heated rats potentially by reducing ischemic injury (secondary to maintaining an adequate MAP). However, the possibility that OT protects against heat-induced ALI by reducing hyperthermia from ~42 °C to ~40 °C cannot be ruled out.

In humans, studies are lacking on the effects of chronic OT administration on blood pressure. However, short-term intravenous administration of OT to women to enhance uterine contraction or decrease blood loss during labor or cesarean delivery decreased blood pressure^43–45^. The hypotensive response to OT is due to decreases in total vascular resistance, stroke volume and cardiac output^46^. On the other hand, plasma OT is related to lower cardiovascular and sympathetic reactivity during public speaking and the forehead cold pressor test in women^47^. In anesthetized rats, OT pretreatment did not reliably increase MAP during pre-ischemia/reperfusion injury, but it did significantly attenuate cardiac ischemia/reperfusion injury^4^. As shown in Fig. 2C,D, OT does not affect either MAP or HR up to 80 minutes. After 100 minutes, both MAP and HR of OT antagonist treated or sham-treated group decline. The changes in hemodynamic markers might be the result of organ failure and not cause of mortality. Moreover, MAP and HR changes of OT antagonist group are comparable with OT heated group. This highly suggests that OT protective effect is independent of hemodynamics. So the beneficial effects of OT on the ischemic lung tissues are likely depended upon direct cytoprotection at the cellular levels. The possible mechanisms, such as involvement of V1a receptor, redistribution of the blood flow and/or hyperthermia in relation to ALI should be emphysized in future experisments.

As for the half time of OT, it may be short and with 60 minutes before heat shock, oxytocin level should be half, and when considering their survival time, the effect of OT may be limited due to its low in concentration. However, according to the findings of Morin *et al*.^48^, a single intravenous bolus of OT was given at a dose of 10000 ng/kg to anesthetized male rats. Blood sample were taken over 72 min to 150 min. The plasma OT concentrations in rats were downed to the ~1000 ng/kg at time “140 min”. Based on their pharmacokinetic properties, in our present study, 80 minutes after an i.v. dose of 20 μg/kg or 20000 ng/kg of OT would have a plasma level of OT at ~2000 ng/kg. Additionally, 2 h after an i.p. dose of OT (0.1 mg/kg) preatment significantly protected against the lipopolysaccharide-induced acute lung injury^17^. Putting these observations together, the dosage of OT used in the present study seems adequate for protecting against heat-induced ALI in rats.

## Conclusion

Anesthetized rats were exposed to OT before the induction of ALI in an experimental heat stroke model. Pretreatment with OT ameliorated heat-induced acute lung edema, neutrophil infiltration, hemorrhage injury and death. However, OT administration during heat exposure did not induce pulmonary protection. The OT receptor antagonist (L-368,899) was administered 10 min before injecting OT to further demonstrate the role of OT in heat-induced ALI, and the results showed that OT could not alleviate ALI and death under these conditions. In conclusion, the present results indicate that OT can reduce the ischemic, inflammatory and oxidative responses related to heat-induced ALI in rats.

## Materials and Methods

### Ethics review and approval

The Institutional Animal Care and Use Committee of Chi Mei Medical Center (Tainan, Taiwan) approved this work, which was performed according to the Institutional and National Ministry of Science and Technology guidelines for laboratory animal care.

### Materials

OT was purchased from Apex Bio (TX, USA). The OT receptor antagonist L-368,899 (purity ≥ 98%) was purchased from Sigma (MO, USA).

### Animals

Male Sprague-Dawley rats (weighing 358–376 g) were purchased from the Animal Resource Center of Chi Mei Medical Center. The animals were housed in a temperature-controlled room with a 12-h light/dark cycle and free access to food and water. The animals were acclimated for at least 1 week before the start of experimentation. In all experiments, adequate anesthesia was maintained by one or more doses of sodium pentobarbital (40 mg/kg body weight, i.p.) to abolish the corneal reflex and pain reflexes induced by tail pinching.

### Assessment of physiological parameters

Blood pressure monitoring and drug treatment were accomplished via the cannulated femoral artery and vein, respectively. A thermocouple and pressure transducer were used to continuously measure the Tco and MAP.

### Induction of heatstroke

Anesthetized rats were exposed to a folded heating pad at 42 °C for 80 min to induce heat stroke, as depicted in Fig. 1. Then, the folded heating pad temperature was kept at ~26 °C. Twenty minutes post heat stress (i.e., 100 min after the start of heat stress), animals displayed heatstroke symptoms of excessive hyperthermia (e.g., Tco: ~42.4 °C) and arterial hypotension (e.g., MAP: ~35 mmHg)^12,18^. We determined the percent survival for all groups.

### Experimental groups

After adaption, all rats were randomly divided into five groups (n = 10 in each group), and all drugs were injected intravenously: (A) control or vehicle-treated nonheated group (V + nonheated; saline 10 min and 60 min before the start of experimentation; 26 °C); (B) V + heated group (saline (1 mL/kg) 10 min and 60 min before the start of heat stress (42 °C for 80 min); (C) OT + heated group (saline 10 min and OT (5 μg/mL/kg, 20 μg/mL/kg or 80 μg/mL/kg) 60 min before the start of heat stress (42 °C for 80 min); (D) L-368,899 + OT + heated group (L-368,899 (5 mg/kg) 10 min and OT (20 μg/mL/kg) 60 min before the start of heat stress (42 °C for 80 min); and (E) heated + OT group (20 μg/mL/kg OT 80 min after the start of heat stress (42 °C for 80 min).

### Lung morphology

The right upper lung was removed, fixed in 10% buffered formalin, embedded in paraffin, cut into 3-μm-thick sections and stained with hematoxylin and eosin (HE). The pathological changes in the lungs were observed using an optical microscope. The histological scoring parameters included edema, neutrophil infiltration and alveolar hemorrhage and were scored by the methods of Matute-Bello *et al*.^49^.

### Lung water content

The severity of lung edema was assessed by the wet weight/dry weight ratio as reported previously^20^. The lung wet weight was measured, and then, the lungs were placed in an oven at 100 °C for 24 h to obtain the dry weight and calculate the wet/dry weight ratio.

### Evans blue dye extravasation assay

The Evans blue dye extravasation assay was conducted by previously described methods^21^. An intravenous dose of 0.2 mL Evans blue dye (4%) was administered to the anesthetized animals 3 h before sacrifice. A spectrophotometer (Thermo Fischer Scientific Inc., Waltham, MA, USA) was used to quantify the amount of extravasated Evans blue dye in the lungs.

### MPO activity in lung tissue

As an indicator of neutrophil infiltration, MPO activity in the ischemic lung tissue was determined as previously described^22^.

### Measurement of cytokines, cellular ischemia and damage markers, and oxidative damage indicators in BALF

The BALF concentrations of cytokines (e.g., TNF-α, IL-1β, IL-6 and IL-10) and indicators of ischemia (e.g., glutamate and lactate/pyruvate ratio), cellular damage (e.g., glycerol), and oxidative damage (e.g., NO metabolites and DHBA) were determined according to previous reports^16,23^.

### Statistical analysis

We performed all statistical data analyses with GraphPad Prism 7.01 (GraphPad Software Inc., CA, USA). Data from multiple independent experiments are expressed as the mean ± standard deviation (SD). Percent survival was compared using Kaplan-Meier analysis followed by the log-rank test. One-way analysis of variance followed Tukey’s multiple comparisons test was performed to analyze differences among multiple groups. Significant differences were established at P < 0.05.

## Data Availability

The corresponding authors will provide data upon reasonable request.
